# From Fibroids to Fasciitis: A Rare Case of Necrotizing Fasciitis Post-Abdominal Hysterectomy

**DOI:** 10.7759/cureus.39758

**Published:** 2023-05-31

**Authors:** Srihita Patibandla, Sania Razzak, Ali Z Ansari, Samuel F Brown

**Affiliations:** 1 Obstetrics and Gynecology, William Carey University College of Osteopathic Medicine, Hattiesburg, USA; 2 Obstetrics and Gynecology, Merit Health Central, Jackson, USA

**Keywords:** proteus mirabilis, leiomyoma, urinary tract infection, uterine fibroid, type ii diabetes, morbid obesity, hysterectomy, necrotizing fasciitis (nf)

## Abstract

Necrotizing fasciitis is a life-threatening bacterial infection characterized by rapid tissue destruction and systemic inflammation. Although it is rare, it can occur at surgical incision sites in procedures such as open abdominal hysterectomy. Prompt diagnosis and treatment are essential to prevent sepsis and multi-organ failure. We present a case of a 39-year-old morbidly obese African American woman with a history of type II diabetes that developed necrotizing fasciitis at a transverse incision site following an abdominal hysterectomy. The infection was complicated by a urinary tract infection caused by *Proteus mirabilis*. Surgical debridement and antibiotic therapy were successfully employed to treat the infection. This case emphasizes the importance of clinical suspicion, early intervention, and appropriate antimicrobial therapy in managing necrotizing fasciitis at incision sites, particularly in patients with additional risk factors.

## Introduction

Necrotizing fasciitis is a rare but potentially life-threatening bacterial infection that can rapidly spread throughout the body [1]. Its incidence in the United States is estimated to be between 500 and 1,000 cases per year, although the actual incidence may be higher due to underdiagnosing and underreporting [2]. The mortality rate for necrotizing fasciitis is high, ranging between 20% and 30%, and early surgical intervention, including aggressive debridement and removal of affected tissue, is critical for improving patient outcomes [3]. Although necrotizing fasciitis can occur in any part of the body, it is rare to see it develop at surgical incision sites, particularly in a patient status post-hysterectomy. However, a few cases have previously been reported in the literature [4,5].

Diagnosing necrotizing fasciitis can be challenging, as it can initially present with nonspecific symptoms. The definitive diagnosis of necrotizing fasciitis relies on clinical suspicion and surgical exploration with biopsy and tissue cultures [1]. Early and aggressive treatment is essential in the management of necrotizing fasciitis, including antibiotic therapy, surgical debridement, and hyperbaric oxygen therapy as an adjunctive treatment for anaerobic infections [4]. In cases of necrotizing fasciitis at surgical incision sites following open hysterectomy, prompt recognition and management are crucial to prevent the development of sepsis and multi-organ failure [4,5]. Early detection and intervention can improve patient outcomes and reduce morbidity and mortality [5]. In this report, we discuss the case of a patient status post-abdominal hysterectomy who developed a necrotizing fasciitis infection which was complicated by morbid obesity and diabetes, in addition to a concomitant urinary tract infection.

## Case presentation

A 39-year-old G1P0010 African American female, body mass index (BMI) of 64.98, with confirmed uterine fibroids and endometriosis visited the clinic for evaluation of debilitating menorrhagia and dysmenorrhea that has been ongoing for several years with menses lasting up to three weeks. The patient has a medical history significant for type II diabetes, hidradenitis suppurativa, and hypertension. Her medications included metformin, lisinopril, and gabapentin. Five years ago, she was started on Mirena intrauterine device (IUD) which lightened up her menses. However, over the past 10 months, her menses started becoming heavier again, lasting eight days at a time, requiring the use of eight pads per day, and causing her to miss work. The patient’s prior endometrial biopsy records obtained from her previous obstetrician/gynecologist (OB/GYN) were negative, and pelvic ultrasound revealed an enlarged uterus with several fibroids. Due to her symptomatic fibroids and history of endometriosis, a hysterectomy was indicated. The patient was treated with Flagyl for a *Trichomonas vaginalis* infection diagnosed via vaginal NuSwab a few weeks prior to her surgery. At the time, the patient’s urine was positive for elevated leukocytes, nitrite, and protein; however, treatment was not yet initiated as the patient was asymptomatic. As a result of her excess weight and lack of visualization of her cervix, she was scheduled for an open total abdominal hysterectomy with bilateral salpingo-oophorectomy and an endometrial biopsy under sedation for the surgery. Her pre-operative glucose levels were within normal limits, her electrocardiogram (EKG) was normal, her hemoglobin and hematocrit were within normal limits, her creatinine was within normal limits, and her chest x-ray was normal. The patient had discontinued her metformin 48 hours before surgery and her lisinopril the day before surgery, and she had been nothing by mouth (NPO) since midnight in preparation for the procedure. She was given a single 2 g dose of prophylactic cefoxitin prior to surgery. The surgery was performed using a Pfannenstiel incision made two fingerbreadths above the pubic symphysis which was extended to 2 cm medial to the anterior superior iliac spine (ASIS) bilaterally. A 26 cm uterus with multiple fibroids was removed per abdomen. The surgery was otherwise uneventful with no complications encountered, and the patient tolerated the procedure well. The wound borders were well-approximated, and the incision was covered with steri-strips and wound dressings. The patient was kept for observation for two days prior to discharge with post-operative cefoxitin, 2 g to be taken every six hours. No Foley catheter was placed.

One week post-operatively, the patient returned to the clinic with a fever, severe pain around the surgical incision site, and inability to ambulate. There was severe tenderness in the lower quadrants at the incision site, although no drainage or signs of infection were present. She was given scripts of amoxicillin-clavulanate and pain medications with instructions to go to the hospital if her condition worsens. She presented to the hospital two days later with worsening symptoms including burning, chills, and inability to control her fever with acetaminophen. Upon examination, the area around the incision and skin of the lower abdomen was edematous and warm to the touch, with serosanguinous discharge present. Computed tomography (CT) of the abdomen and pelvis revealed inflammatory changes in the anterior abdominal wall involving the panniculus with ill-defined fluid and multiple air bubbles present. Urinalysis revealed 3+ blood, 3+ protein, and 2+ leukocyte esterase. Hemoglobin A1c (HgbA1c) level was 7.0%, and bedside glucose was 185 mg/dL; the patient’s blood sugar levels were managed in the hospital with an insulin sliding scale. Blood culture was negative, but urine culture was positive for gram-negative rods and diphtheroids. Additionally, leukocytosis, elevated creatinine, procalcitonin, lactic acidosis, fever, and tachycardia suggested sepsis secondary to urinary tract infection and possible panniculitis. Despite treatment with IV antibiotics and topical Neosporin, the patient’s white blood cell (WBC) count continued to increase, and she was transferred to another facility for surgical debridement and drainage.

**Figure 1:**
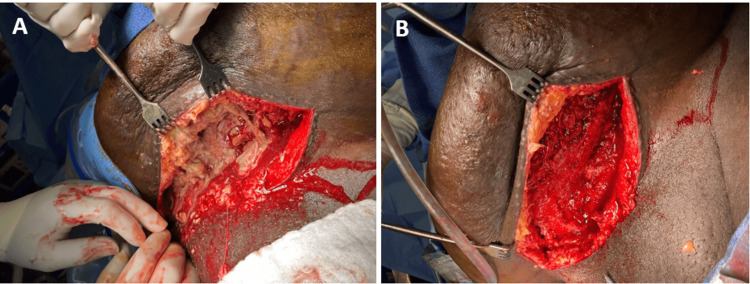
Intraoperative photographs of the surgical site. (A) Before and (B) after completion of surgical debridement.

The patient was NPO since midnight in preparation for the procedure and was given a 2 g dose of prophylactic cefoxitin before surgery. During the surgery, a scalpel was used to open the Pfannenstiel incision site to the depth of the subcutaneous tissue, and the patient was subsequently diagnosed with necrotizing fasciitis. Serosanguinous fluid build-up and necrotic tissue were noted. Non-viable tissue was thoroughly debrided, the area was irrigated with vancomycin-infused normal saline, and a wound vacuum-assisted closure (VAC) was placed at the incision site to allow healing from the inside (Figures 1A, 1B). The patient was treated empirically with post-operative vancomycin, Zosyn, and clindamycin until wound culture results returned. The wound culture obtained during surgery was positive for *Proteus mirabilis*, and the patient’s antibiotics were changed to cefazolin. The patient tolerated the procedure well, and her post-operative glucose level was 121 mg/dL. No Foley catheter was placed. The patient had a moderate Wells score of 2 as she has limited mobility secondary to her body habitus. She was given 2.5 mg apixaban twice daily for DVT prophylaxis, and ambulation was encouraged prior to discharge with home health for peripherally inserted central catheter (PICC) Line IV antibiotics. Hemodynamic stability was maintained through IV fluids. Prior to discharge on post-operative day four, the patient was stable, and her WBCs decreased with signs of infection resolving throughout the post-operative course. The wound was clean and dry with no discharge or signs of infection present, and the abdomen remained tender. The patient followed up in the clinic two weeks post-operatively and reported significant symptomatic improvement. Reconstructive surgery was not deemed necessary for the patient as the wound had approximated well and was healing appropriately after the removal of the wound VAC.

## Discussion

Necrotizing fasciitis is a rare and life-threatening soft tissue infection caused by a variety of bacterial pathogens, with group A streptococcus being the most common [6]. Other organisms associated with necrotizing fasciitis include *Staphylococcus aureus*, *Klebsiella pneumoniae*, *Clostridium perfringens*, and *Vibrio vulnificus*, among others [7,8]. The bacteria invade the subcutaneous tissue and fascia, typically through a breach of the skin, leading to tissue destruction and production of toxins that cause necrosis and systemic inflammatory response syndrome (SIRS) [3,9]. The early signs of necrotizing fasciitis can be subtle and non-specific, but typically include severe pain at the incision site, fever, erythema, and edema [2,10,11]. As the infection progresses, bullae or crepitus may develop, and the patient may develop sepsis and multi-organ failure if not treated early [8].

The definitive diagnosis of necrotizing fasciitis relies on clinic suspicion and surgical exploration with biopsy and tissue cultures [12]. Imaging studies such as CT scans, ultrasound, or MRI may aid in the diagnosis but are not definitive [11,12]. In the laboratory, elevated WBC count, elevated creatinine kinase levels, and metabolic acidosis may be seen [11,12]. However, the sensitivity and specificity of these laboratory tests are low, and they should not be relied upon to exclude the diagnosis of necrotizing fasciitis [11]. The most critical factor in the diagnosis of necrotizing fasciitis is a high degree of clinical suspicion, especially in patients like ours with risk factors such as immunosuppression, obesity, diabetes, or recent surgery [13]. Diabetes compromises the immune system, impairs wound healing, and promotes bacterial growth, leaving patients more vulnerable to infections. Peripheral neuropathy and vascular disease which are commonly associated with diabetes can allow the progression of necrotizing fasciitis [13,14]. Morbid obesity, characterized by an elevated BMI, can impair blood circulation, can compromise the immune system, and creates moist areas for bacterial growth [2,13]. Managing necrotizing fasciitis in these patients requires recognizing these risk factors, taking preventative measures, and early intervention. For our patient, preventative measures taken prior to surgery included EKG testing, chest x-ray, optimizing glycemic control, and testing hemoglobin, hematocrit and creatinine levels. In addition, post-operative wound care was discussed with the patient.

Treatment for necrotizing fasciitis involves a combination of surgical debridement, streamlined antibiotic therapy, and occasionally, hyperbaric oxygen therapy. The primary treatment is surgical debridement, involving thorough removal of necrotic tissue and exploring the affected area to identify the depth of the infection [15,16]. Debridement should be performed as early as possible to stop the progression of the infection as it could lead to sepsis and multi-organ failure. Empirical broad-spectrum antibiotic therapy should be started immediately, targeting both aerobic and anaerobic microorganisms. The antibiotics can be given in combination; for example, a regimen of penicillin, clindamycin, and an aminoglycoside can be given to cover both gram-positive and gram-negative bacteria [6,15,16]. In the case of the patient presented, empiric antibiotics included vancomycin, clindamycin, and Zosyn. These antibiotics can be adjusted once wound culture results are obtained as done for our patient whose antibiotics were changed to cefazolin to treat *Proteus mirabilis*. Hemodynamic stability should be maintained through aggressive fluid resuscitation and monitoring of vital signs [16]. Supportive care such as pain management, wound care, and dietary monitoring are also crucial [16]. Hyperbaric oxygen therapy has been suggested for necrotizing fasciitis to create a high-pressure oxygen environment that enhances tissue oxygenation, promotes wound healing, and prevents the growth of anaerobic bacteria. This was not used for our patient as we did not have a hyperbaric chamber at our facility.

In the case report, the patient was a morbidly obese African American woman with a history of type II diabetes and had undergone a total abdominal hysterectomy. A Pfannenstiel incision is a transverse abdominal incision made two finger widths above the pubic symphysis that is commonly used for cesarean sections and gynecologic surgeries. While necrotizing fasciitis is rare, a few cases have previously been reported following gynecologic procedures done using transverse incisions [4,5]. A study published in the *Journal Surgical Endoscopy* discusses how laparoscopic surgery is associated with a protective effect against surgical site infections compared to open surgery [17]. The authors proposed that this is likely due to contributing factors such as a shorter surgical incision, decreased tissue trauma and contamination, and elimination of mechanical retraction of the abdominal wall [17]. However, more research is needed to fully understand the risk factors associated with necrotizing fasciitis following different types of surgical incisions.

The case of the patient presented here is unique as the wound culture obtained during surgical debridement was positive for *Proteus mirabilis*, a gram-negative rod that is commonly found in the gastrointestinal and genitourinary tracts [18]. Additionally, the patient’s urine culture was positive for gram-negative rods, indicating the presence of a urinary tract infection. A 2008 study published in *International Orthopedics* found that a statistically significant percentage of patients with a pre-operative urinary tract infection (UTI) had some form of post-operative delay in surgical wound healing or confirmed infection following orthopedic arthroplasty [19]. While possible that the pre-operative UTI is a confounding factor caused by poor physiological status, this seems unlikely given that all patients were of a similar age group and there was no statistically significant link between UTI and other comorbidities [19]. As *Proteus mirabilis* is one of the leading causes of UTIs, the urinary tract is likely the source of the bacteria that caused the necrotizing fasciitis. While *Staphylococcus aureus* and Group A Streptococcus are the most common pathogens associated with necrotizing fasciitis, other bacteria such as *Proteus mirabilis* have been reported in the literature [9,10]. This case highlights the importance of obtaining wound cultures and urine cultures in patients with suspected necrotizing fasciitis, as it may provide important information for guiding antimicrobial therapy.

## Conclusions

In conclusion, necrotizing fasciitis is a rare but potentially life-threatening bacterial infection that can rapidly spread throughout the body. This case report demonstrates the unique presentation of necrotizing fasciitis in an overweight patient following a total abdominal hysterectomy. The complications were likely due to the patient’s morbid obesity, which required an open procedure, and the presence of a urinary tract infection. Prompt recognition and management of necrotizing fasciitis at incision sites are crucial to improve patient outcomes and reduce morbidity and mortality associated with the infection. A high degree of clinical suspicion, aggressive surgical intervention, and antimicrobial therapy are the mainstay of treatment.
